# Fuzzy controller-driven pattern search optimization for a DC–DC boost converter to enhance photovoltaic MPPT performance

**DOI:** 10.1038/s41598-025-16255-3

**Published:** 2025-09-05

**Authors:** Maher G. M. Abdolrasol, Sieh Kiong Tiong, Pin Jern Ker, Shaheer Ansari, Afida Ayob, Hassan Abdurrahman Shuaibu, Ahmed Said Al Busaidi, Taha Selim Ustun

**Affiliations:** 1 Institute of Sustainable Energy, Universiti Tenaga Nasional, Kajang, 43000 Malaysia; 2 Faculty of Engineering and Technology, Sunway University, Bandar Sunway, Petaling Jaya, 47500 Malaysia; 3 Departments of Electric, Electronics and Sys. Eng., Universiti Kebangsaan Malaysia, Bangi, 43600 Malaysia; 4 Department of Electrical, Telecommunications and Computer Engineering, Kampala International University, Kampala, Uganda; 5 College of Engineering and Architecture, University of Nizwa, Nizwa, Oman; 6 Fukushima Renewable Energy Institute, AIST (FREA), Koriyama, 963-0298 Japan

**Keywords:** Photovoltaic, MPPT, Optimal fuzzy controller, Pattern search optimization, DC-DC boost converter, Renewable energy, Energy infrastructure, Renewable energy, Electrical and electronic engineering

## Abstract

This article demonstrates maximum power point tracking (MPPT) using a DC-DC boost converter. It introduces an intelligent control technique with fuzzy-based pattern search (PS) optimization for the MPPT controller, enhancing energy conversion efficiency. The fuzzy-PS approach is further refined with PA optimization. A comprehensive performance evaluation compares it with various optimization algorithms. The controller is tested under changes in irradiance and temperature, showing its performance against the Perturb and Observe (P&O) algorithm. The fuzzy controller is optimized to provide the best membership functions (MFs) using PS optimization, particle swarm optimization (PSO), and genetic algorithm (GA), with root mean square error (RMSE) as the objective function. PS optimization outperforms other algorithms. The fuzzy-PS optimization achieves the lowest RMSE of 0.6861 after 100 iterations, while fuzzy-GA and fuzzy-PSO reach RMSEs of 1.257 and 0.9454, respectively. The proposed fuzzy-PS MPPT controller effectively adapts to irradiance and temperature variations, achieving maximum power outputs up to 74.48 kW and Comparative evaluations revealed an average MPPT efficiency of 99.7%, demonstrating superior tracking performance compared to the P&O algorithm.

## Introduction

The maximum power point tracking (MPPT) maximizes the efficiency of solar photovoltaic (PV) systems. MPPT algorithms ensure that the PV panels operate at their maximum power output by continuously tracking and adjusting the operating point to match the varying environmental conditions. This technology is essential for optimizing energy harvest, improving system performance, and enhancing the overall economics of solar power generation. The MPPT algorithm employs a perturbation-and-observation (P&O) method, adjusting the reference voltage based on changes in power and voltage. By incorporating decision-making processes, the algorithm ensures the reference voltage remains within specified limits, providing a robust solution for tracking the maximum power point in a PV system. Visualizing this algorithm aids in understanding and facilitates the implementation of efficient MPPT in a DC-DC boost converter. The method involves sensing the voltage and current from the photovoltaic (PV) output, utilizing these values to implement MPPT, and obtaining the required duty ratio for pulse width modulation (PWM) generation, which is then applied to the gate terminals of the insulated-gate bipolar transistor (IGBT)^1^. On the other hand, the popularity and diverse applications of fuzzy logic have considerably grown, encompassing consumer products, industrial process control, and medical instrumentation, making it a better option to replace the P&O method. Fuzzy logic, in its broad sense, involves the theory of fuzzy sets, where membership in a set is a matter of degree rather than having well-defined boundaries. The method discussed in this article begins by sensing the voltage and current from the PV terminals. Instead of determining the duty ratio, we derive a reference voltage as the output. This reference voltage is then compared with the actual voltage to generate an error signal, which is subsequently fed into a fuzzy logic controller (FLC)^2,3^. The output of the FLC provides the required duty ratio for PWM generation. This duty ratio is subsequently compared with a carrier signal, and the resulting PWM is applied to the gate terminal of the IGBT diode^4^.

In literature^5–10^, various MPPT algorithms address issues like mismatched non-uniform isolation, reducing PV output power, and causing potential damage to PV cells from hot spots. Given the time-varying dynamics of PV systems under partial shading, effective MPPT designs should track the global maximum power point under different conditions and adapt to changes in PV array characteristics^11^. MPPT techniques such as hill climbing perturb and observe, and incremental conductance aim to enhance PV system efficiency. The hill climbing (HC) method perturbs the duty ratio of the power converter^12^, and the perturb and observe (P&O) method perturbs the operating voltage of the PV system^13^. Unfortunately, both methods exhibit oscillations at the MPP due to continuous bidirectional changes in perturbation, resulting in power losses. Several MPPT techniques, including hill climbing, perturb and observe, and incremental conductance, have been suggested to enhance the efficiency of PV systems. The HC method introduces a perturbation in the duty ratio of the power converter, while the P&O method perturbs the operating voltage of the PV system^14^. Unfortunately, both methods exhibit oscillations at the MPP due to the continuous bidirectional changes in perturbation aimed at maintaining the MPP, leading to power losses.

In the quest to optimize photovoltaic system efficiency, various MPPT algorithms have been explored, each with distinct advantages and challenges. The P&O algorithm, though independent and less complex, oscillates around the MPP and struggles with fast changes. HC is easy to implement but may lose precision during large irradiance changes^15^. Incremental Conductance (IC) contends with oscillations, power losses, and higher computational burden^16^. artificial neural network (ANN) offers fast convergence but needs accurate weight determination, risking local minima trapping^17^. constriction factor-inertia weight algorithms are effective but prone to local minima and premature convergence^18–20^. FLC avoids a mathematical model but requires time-consuming tuning^21–23^. This literature explores these MPPT techniques, illuminating their attributes and limitations in advancing photovoltaic system performance. Table 1 List of major advantages and disadvantages of the popular MPPT algorithms. This paper introduces an intelligent control technique that utilizes fuzzy-based pattern search (PS) optimization for the MPPT controller, aiming to enhance energy conversion efficiency. The initial fuzzy-PS approach is further refined through the integration of PS. A comprehensive performance evaluation is conducted by comparing it with various optimization algorithms to assess its effectiveness and compare it against their respective performances.

**Table 1:** List of major advantages and disadvantages of the popular MPPT algorithms.

MPPT type	Advantages	Shortcoming
P&O	Not dependent on the PV array Simpler to implement	Exhibits oscillations around the MPP Unsuitable for rapid changes Performance is heavily reliant on the fixed step of Δv/Δi.
IC	Not dependent on the PV array Moderately complex to implement	Experiences slight oscillations around the MPP Incurs power losses due to noise and measurement errors Involves a higher computational burden when compared to the P&O method
HC	Simple to implement Not reliant on the PV array	Susceptible to failure with substantial irradiance changes Lack of guaranteed Maximum Power Point (MPP) precision Results in low extracted energy
CI	Demonstrates relative effectiveness in achieving a precise solution	Susceptibility to getting trapped in local minima Risk of premature convergence
ANN	Demonstrates a rapid convergence speed Proves to be relatively effective for non-linear systems	Accurate determination of weights for neurons requires a precise training process Possibility of getting trapped in local minima
Fuzzy	Operates without the need for a mathematical model of the system Demonstrates effectiveness, especially in dealing with non-linear systems	The process of tuning the fuzzy system is time-consuming

In this study, the fuzzy MPPT method is employed to address the shortcomings of the conventional MPPT fuzzy method. An optimization process is utilized to fine-tune the membership functions (MFs) and overcome the challenges associated with trial and error^3,24–29^. The primary contribution of this paper lies in finding the optimal fuzzy solution by employing various optimization algorithms to compare their effectiveness. The goal is to determine which optimization method provides the best solution and optimal output by tuning key parameters for superior tracking performance. Three optimization algorithms, namely PS, genetic algorithm (GA), and particle swarm optimization (PSO), were employed^30^. However, in this comparative analysis, PA demonstrated the best performance, achieving a minimum objective within 100 iterations in this optimization. It is noteworthy that other parameters remained constant across all optimization methods using MATLAB R2023b.

A critical observation from the A assessment of the current MPPT algorithms identifies a number of issues that spur the current investigation. Despite being straightforward and popular, traditional techniques like P&O and hill climbing have lower performance under quickly changing climatic circumstances and continuous oscillations around the maximum power point. Though they are computationally demanding and prone to premature convergence or entrapment in small minima, metaheuristic optimizers like GA and PSO enhance tracking. Despite being model-free and efficient for nonlinear systems, fuzzy logic controllers necessitate a great deal of human membership function and rule tuning, which is frequently laborious and unsatisfactory. Additionally, by striking a balance between exploration and exploitation without requiring gradient information, derivative-free local optimization techniques like pattern search offer viable substitutes that improve convergence and resilience. By using pattern search to optimize the fuzzy controller parameters, tuning issues can be resolved and MPPT efficiency can be increased under temperature and irradiance variations^31^. The development described in this study is therefore motivated by the fact that the combination of fuzzy logic and pattern search optimization fills important holes in current MPPT tactics.

The main contributions of this paper are summarized as follows:

- Development of a fuzzy logic-based MPPT controller for a DC-DC boost converter tailored to photovoltaic systems.
- Implementation and integration of PS optimization to automatically tune the fuzzy controller’s membership functions, improving tracking accuracy without requiring gradient information.
- Comprehensive comparative analysis of three optimization algorithms PS, GA, and PSO to identify the most effective tuning method for MPPT.
- Demonstration of the proposed fuzzy-PS controller’s superior performance under varying irradiance and temperature conditions, with quantitative results validating improved power tracking and reduced oscillations.
- Provision of detailed simulation results highlighting the robustness and adaptability of the optimized controller, showcasing its potential for real-world PV system applications.

## System configuration

**Fig. 1:**
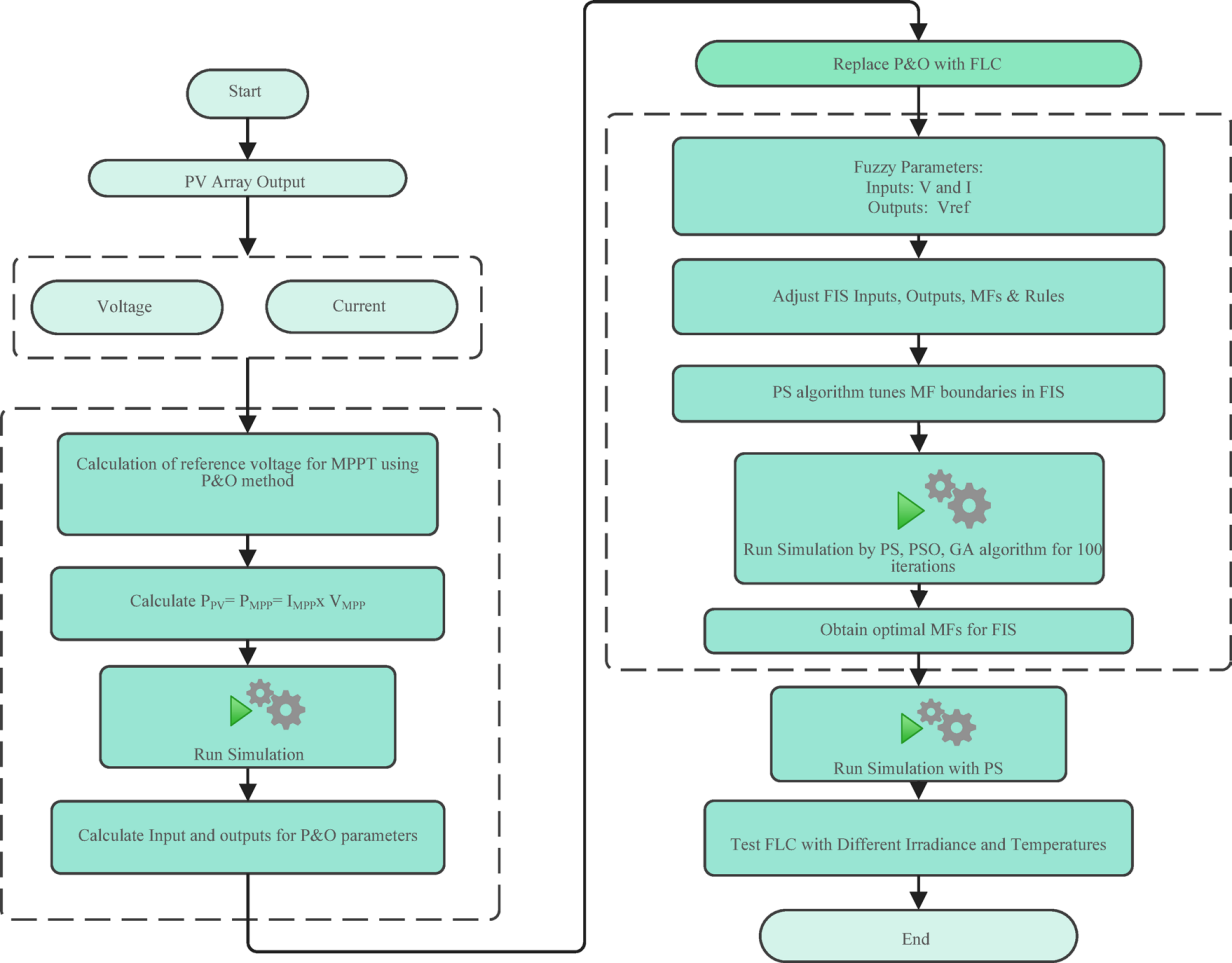
Flowchart for system configuration and optimization steps for the proposed PV-MPPT system, transition from a conventional P&O method to the optimized fuzzy logic controller.

To illustrate the implementation and optimization process of the proposed MPPT control system, Fig. 1 presents the complete workflow from initial PV array output to the deployment and testing of the optimized fuzzy logic controller. The flow chart clearly outlines the transition from conventional P&O-based MPPT to the advanced fuzzy controller optimized by PS and other algorithms, as well as the steps for parameter tuning, simulation, and performance evaluation under various environmental conditions.

## Boost converter circuit

**Fig. 2:**
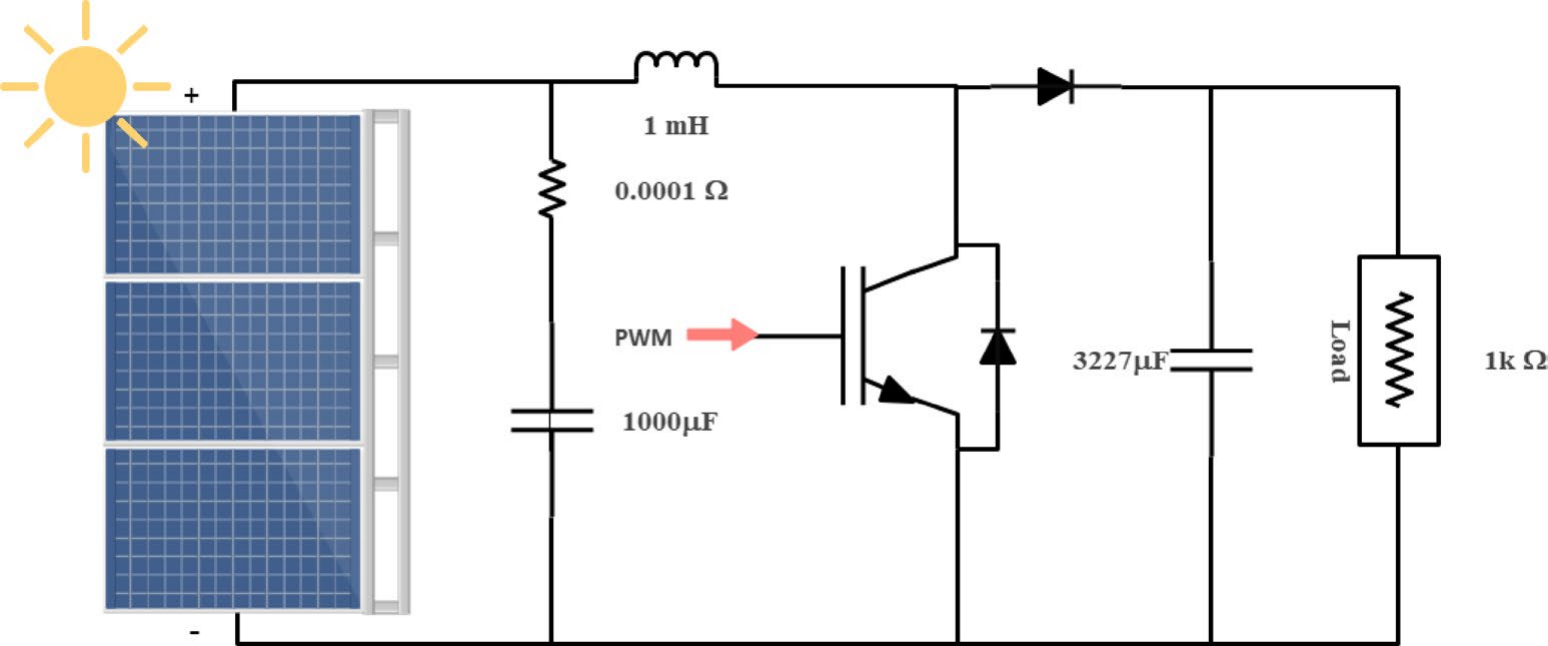
Boost converter design with photovoltaic and load.

In PV energy conversion systems, the DC-DC boost converter is essential for matching the PV array output to the system voltage requirements and for enabling fast, accurate MPPT. Its performance directly impacts the overall efficiency, response speed, and reliability of the PV system, especially under dynamic environmental condition. Figure 2 shows the boost converter circuit operating within predetermined parameters and voltage ranges. The PV system can manage a rated power of 100KW and has an output voltage of 600 V and an input voltage range of 250 V to 350 V. The inductance (L) is set at 1.45mH, and the capacitance is specified as 3227µF calculated based on Eqs. (1) and (2)^32,33^.

1 $$\:{L}_{\text{i}\text{n}\text{d}\text{u}\text{c}\text{t}\text{a}\text{n}\text{c}\text{e}}\:=\frac{{V}_{input\:}({V}_{output\:}-{V}_{input\:})\:\:}{{f}_{sw}*\varDelta\:I*{V}_{output\:}}$$

2 $$\:{C}_{\text{c}\text{a}\text{p}\text{a}\text{c}\text{i}\text{t}\text{a}\text{n}\text{c}\text{e}\:}\:=\frac{{V}_{output\:}({V}_{output\:}-{V}_{input\:})\:\:}{{f}_{sw}*\varDelta\:V*{V}_{output\:}}$$

In detail, the input voltage ranges from 250 V to 380 V, while the output voltage is maintained at a constant 600 V. The rated power of the system is 100KW, with a switching frequency ($\:{f}_{sw}$) of 5 KHz^32,34^. To ensure stable operation, the boost converter circuit incorporates a 5% current ripple (ΔI) and a 1% voltage ripple (ΔV). With these parameters, the input current is calculated as 400 A, considering the rated power and input voltage. The corresponding current ripple is determined as 20 A, and the voltage ripple is found to be 6 V. The output current, derived from the rated power and output voltage, is established at 166 A. The inductance (L) and capacitance (C) values are calculated using the provided formulas, In practice, non-idealities such as core losses and equivalent series resistance were considered during component selection, further supporting the converter’s stable operation and integration with the proposed fuzzy-PS MPPT strategy. Simulation confirms efficiency above 97% and rapid transient response, which are critical for effective MPPT during fast irradiance or load changes^35^.

## Photovoltaic array configurations

**Fig. 3:**
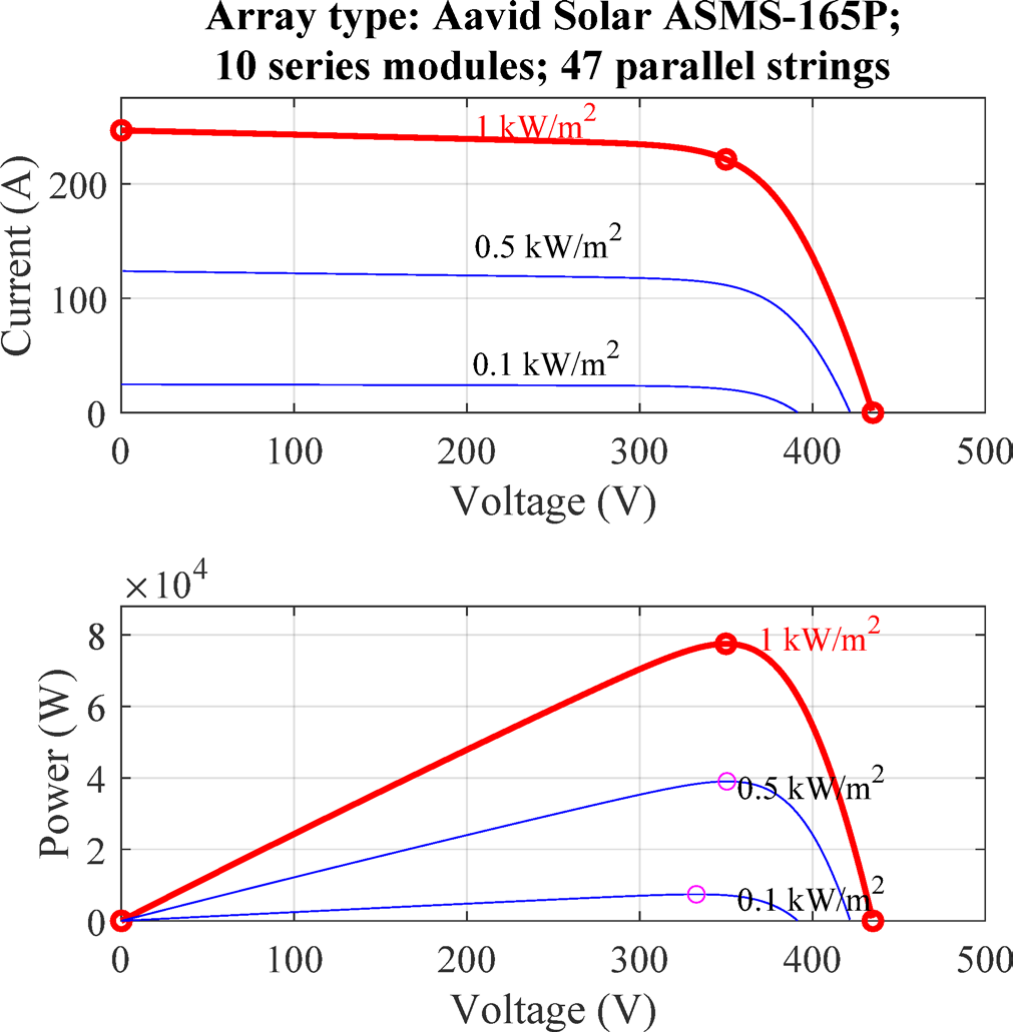
Irradiance levels for Aavid Solar ASMS-165P array (10 series modules, 47 parallel strings).

The photovoltaic (PV) array employed in this study is configured to ensure high power capacity and operational flexibility under varying environmental conditions. The array architecture consists of multiple parallel strings, with each string composed of PV modules connected in series. This design enables the system to achieve both the desired voltage and current ratings while maximizing energy yield. To model and simulate the PV system, the commercially available Aavid Solar ASMS-165P module was selected, based on data provided by the System Advisor Model (SAM) developed by the National Renewable Energy Laboratory (NREL)^36^. The array specifications include 47 parallel strings, with 10 modules linked within each string in series. Table 2 presents the key electrical and thermal specifications of the ASMS-165P PV module, including its power output, voltage and current characteristics, and temperature coefficients, and Fig. 3 illustrates the irradiance levels experienced by the full PV array configuration, specifically depicting the performance of 10 series modules in each of the 47 parallel strings. This PV array configuration was selected to ensure the system meets the overall rated power requirement of 100 kW, while allowing for detailed analysis of array performance under realistic operating conditions. The sizing and electrical characteristics of the array are fully integrated within the simulation model, providing an accurate platform to evaluate the effectiveness of the proposed MPPT control strategy and the dynamic response of the boost converter circuit under varying irradiance and temperature scenarios.

**Table 2:** Hardware PV module specifications for ASMS-165P.

Parameters	Data
Power output under standard test conditions (W)	165
Power output under standardized conditions used for testing (W)	146.3
Bifacial	No
Power density under standard test conditions (W/m^2^)	126.923
Density of power under PTC conditions (W/m^2^)	112.538
Voltage at maximum power (V)	35.0
Current at maximum power (A)	4.71
Open-circuit voltage (V)	43.5
Short-circuit current (A)	5.25
Nominal operating cell temperature (°C)	45.0
Temperature coefficient of open circuit voltage. (%/°C)	− 0.393
Temperature coefficient of short-circuit current (%/°C)	0.03
Temperature coefficient of maximum power (%/°C)	− 0.519

## MPPT control design

**Fig. 4:**
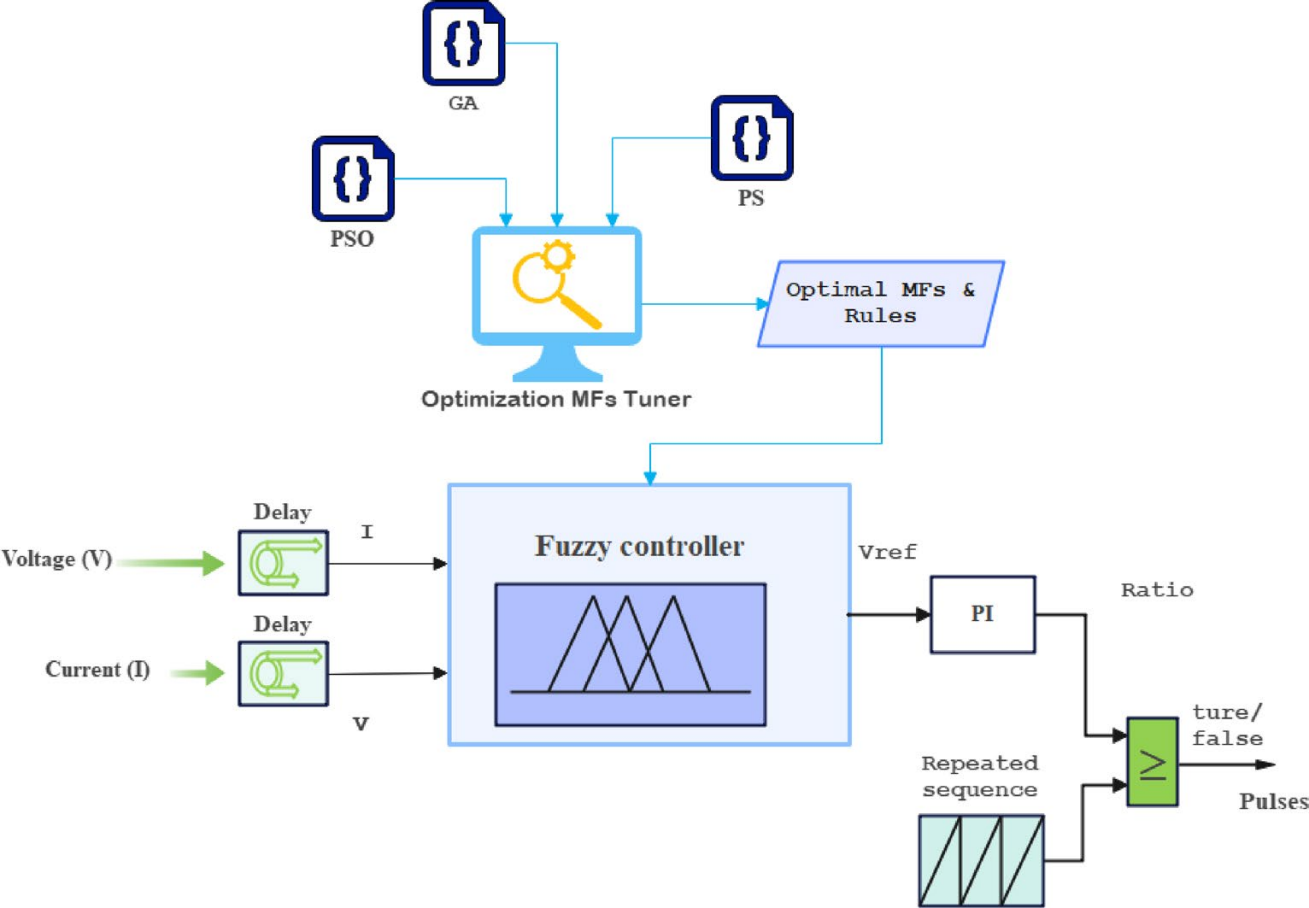
Controller design flowchart for the fuzzy logic and optimization-based MPPT algorithms.

In this study, the MPPT control strategy is developed and implemented for a PV system to ensure optimal power extraction under dynamic environmental conditions. Initially, the widely used perturb and observe (P&O) algorithm^37,38^ was adopted and implemented in MATLAB to serve as a benchmark for controller development. The P&O method offers advantages such as simplicity, easy implementation, and independence from the PV array parameters. However, it suffers from inherent limitations, including steady-state oscillation around the maximum power point (MPP), reduced accuracy during rapidly changing irradiance, and performance dependence on the choice of perturbation step size of Δv/Δi. To address these drawbacks, input-output datasets were collected from the P&O-based MPPT operation as a reference for developing and optimising advanced fuzzy logic controllers combined with metaheuristic optimization algorithms such as GA, PSO, and PS. These optimization techniques are employed to fine-tune the membership functions and rule base of the fuzzy controller, thereby improving tracking accuracy, convergence speed, and robustness to environmental disturbances. Figure 4 presents the controller design flow, including the integration of fuzzy logic and optimization algorithms, illustrating the signal flow and the decision-making process in the proposed MPPT strategy. The MPPT algorithm seeks to track the MPP by adjusting the duty cycle of the DC-DC converter to ensure that the product of V and I is maximized, typically by comparing it to the reference voltage $\:{V}_{ref}$. adjust the duty cycle and track the MPP under varying environmental conditions perturbation as in (3), observation as in (4) For comparison use Eq. (5) this will show the update for the power.

3 $$\:{V}_{new}={V}_{n-1}+\varDelta\:V$$

4 $$\:{P}_{current}={V}_{new}*{I}_{current}$$

5 $$\:If\:\:{P}_{current}>{P}_{previous\:}\:\:update\:\left\{\begin{array}{c}{V}_{new}={V}_{n-1}+\varDelta\:V\\\:or\\\:{V}_{new}={V}_{n-1}-\varDelta\:V\end{array}\right.\:$$

## Fuzzy based optimization algorithms

An optimization approach for enhancing the performance of standalone PV boost converters is introduced. Employing optimization to find the best parameters for the fuzzy logic controller is essential and a smart solution. A developed computational intelligence-based technique is adept at addressing both single and multimodal optimization challenges. The application of optimization is anticipated to elevate the effectiveness of fuzzy logic controllers in PV boost converters. The optimization process involves adjusting the MFs of an FLC to suit the MPPT controller output, with the root mean squared error (RMSE) of the output ratio serving as the objective function. The system is simulated in the MATLAB R2023b environment to present the performance of the proposed MPPT boost converter controller. Considering the non-linearity of the MPPT power output process in a PV boost converter, fuzzy logic is a convenient method to adopt in an MPPT control for a boost converter. The FLC represents the human expert decision in the problem-solving mechanism.

While various optimization algorithms such as GA, PSO, and Differential Evolution (DE) are commonly used for tuning fuzzy logic controllers, PS optimization was selected in this study due to several advantages. PS is a direct search method that does not require gradient information, making it highly robust for optimizing non-differentiable or complex objective functions, which are typical in fuzzy controller design. Compared to population-based algorithms, PS generally offers faster convergence, lower computational overhead, and is easier to implement for problems with a moderate number of variables. These benefits make PS particularly suitable for real-time and embedded applications where computational efficiency and reliability are essential. For comparison, we use two other optimization algorithms: GA and PSO. The genetic algorithm is a population-based global optimization method that employs random search through mutation and crossover operations among its population members. Additionally, PSO is also a population-based global optimization method, where members traverse the search region in a coordinated manner.

Tuning methods always require a global optimization Toolbox software for easy for user. For all global optimization Toolbox optimization methods, in the Fuzzy Inference System (FIS) tuning options. The setup for the approach includes the maximum number of rules, denoted as the number of rules, in a FIS following optimization is determined when employing the learning optimization type. It is important to highlight that the actual number of rules in the optimized FIS may be less than a number of rules due to the removal of duplicate rules sharing identical antecedent values during the tuning process. Then select a specific method to set the seed for the random number generator before initiating the tuning process. Initialize the generator with seed zero. Initialize the generator with a seed of zero. The chosen distance metric for calculating the cost related to optimized parameter values concerning training data is specified as RMSE. This entails computing the root-mean-squared error and selecting this parameter to invalidate any generated parameter values during the tuning process. To streamline the configuration of the number of rules based on the count of input variables and the number of MFs assigned to each input variable.

## Pattern search optimization

The underlying principles of the PS are involved in iteratively exploring a search space based on directional patterns, evaluating solutions, and adjusting the search direction to converge towards an optimal solution while avoiding local optima given the problem. It belongs to the class of direct search or derivative-free optimization methods^39,40^. The principle is to iteratively explore the solution space by adjusting the search pattern and updating the current solution. The algorithm keeps running until a halting requirement is satisfied or a workable solution is discovered. PS optimization offers advantages such as being derivative-free, making it applicable to problems without gradient information, and its suitability for global optimization in non-convex, multimodal scenarios^41^. The method is robust and can handle a variety of optimization problems effectively, striking a balance between exploration and exploitation in complex solution spaces. It demonstrates convergence even in the presence of noise or uncertainties. However, there are notable disadvantages. The computational cost of gradient-based methods can be high due to the large number of function evaluations and slower convergence rate. Sensitivity to the initial guess and limitations in scalability, especially in high-dimensional spaces, are challenges. Additionally, there might be a lack of strong theoretical guarantees, particularly for certain types of objective functions. Careful consideration of these factors is crucial in selecting pattern search optimization for specific optimization problems.

**Fig. 5:**
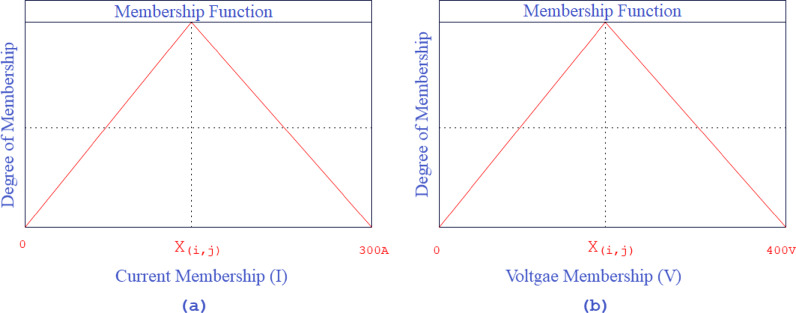
Degree and ranges of membership functions.

Pattern search involves evaluating the objective function at points in a mesh. The size of the mesh can influence the speed of the solution however, the size can be controlled of the mesh using options. The following are the steps of the PS optimization algorithm for optimizing the fuzzy controller. The voltage and current input membership function ranges and degrees are displayed in Fig. 5.

### Initialization

Start with the initial values of MF limitations for the initial search pattern and initial mesh size. The mesh at each iteration is the span of a set of search directions that are added to the current point, scaled by the current mesh size, which starts with an initial mesh size set in the InitialMeshSize. The limits of each population in $\:{L}_{(\text{i},\text{j})\:}$ is updated according to the following expression,

6 $$\:{X}_{i,j}^{k+1}={X}_{i,j}^{k}+{\alpha\:}_{i,j}^{k+1}*{d}_{i,j}^{k}$$

Where: $\:{X}_{i,j}^{k}$ is the current point in the parameter space, $\:{\alpha\:}_{i,j}^{k+1}$ is the step size, ​$\:{d}_{i,j}^{k}$ ​is the search direction and $\:{X}_{i,j}^{k+1}$ is the next point.

### Pattern scaling and mesh accelerator

Assessing the objective function at the current MFs boundaries, Mesh Scaling is employed to enhance the minimization of poorly scaled optimization problems. Scaling involves rotating the pattern by a certain degree and scaling along the search directions. The ScaleMesh option is deactivated if the problem is well-scaled. For this particular problem, set ScaleMesh to false since the contest represents a well-scaled objective function.

Pattern search quickly finds the vicinity of an optimal point but can be slow. The Search Function enables optional searches before polling at each iteration, improving efficiency. PS generates search points by evenly spacing them within specified bounds. Pattern search algorithms utilize the Armijo-Goldstein condition to direct their search, ensuring that the step size effectively decreases the objective function value as anticipated.

7 $$\:f\left({X}_{i,j}^{k+1}\right)\le\:f\left({X}_{i,j}^{k}\right)+c*{\alpha\:}_{i,j}^{k+1}*\nabla\:f\left({X}_{i,j}^{k}\right)*{d}_{i,j}^{k}$$

Where: $\:f\left({X}_{i,j}^{k}\right)$ is the objective function value at the current point $\:{X}_{i,j}^{k}$, $\:\nabla\:f\left({X}_{i,j}^{k}\right)\:$is the gradient of the $\:{X}_{i,j}^{k+1}$ objective function at $\:{X}_{i,j}^{k}$, c is a parameter which controls the decrease in the objective function value between 0 and 1.

### Pattern polling and convergence check

During each step, the algorithm evaluates objective function values for points in the current mesh. polling stops when a point with a lower objective function value than the current one is found. This successful poll makes the newly found point the current point in the next iteration till the end. If no improvement is found, the poll is unsuccessful, and the current point remains unchanged in the next iteration. With the Complete poll set to On, the algorithm computes objective function values for all mesh points. A successful poll occurs if the mesh point with the smallest value is lower than the current point as in (8).

8 $$\:\frac{{\left| {f\left( {X_{{i,j}}^{k} } \right) - f\left( {X_{{i,j}}^{{k - 1}} } \right)} \right|}}{{\left| {f\left( {X_{{i,j}}^{k} } \right)} \right|}} < \upvarepsilon$$

Where Є is a small positive threshold value, typically representing a tolerance level for convergence. After polling, the algorithm changes the value of the mesh size $\:{\varDelta\:}^{m}$. The default is to multiply$\:\:{\varDelta\:}^{m}$. by 2 after a successful poll, and by $\:1/2$ after an unsuccessful poll as in Eq. (9).

**Fig. 6:**
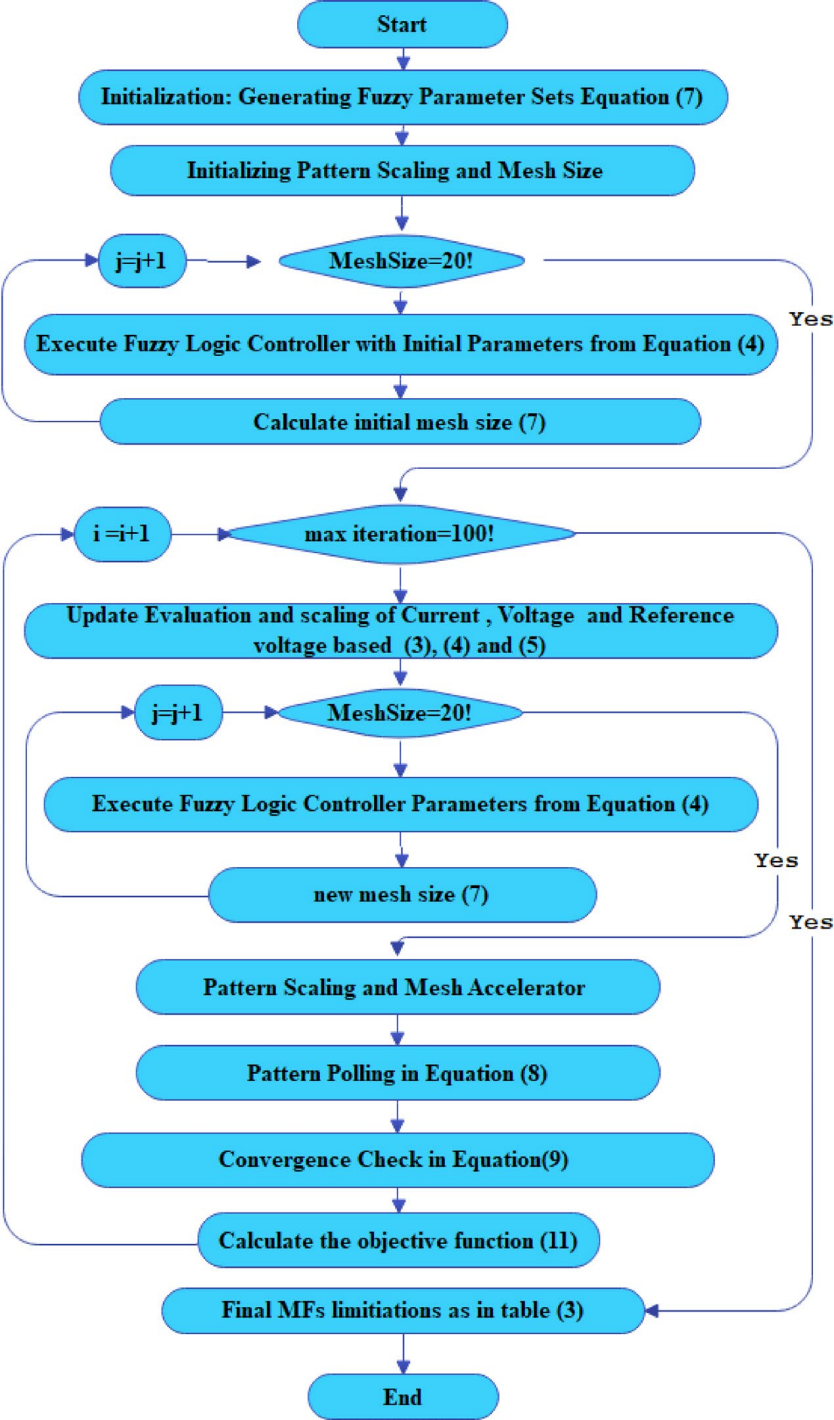
Schematic of the pattern search optimization algorithm utilized for optimizing membership functions parameters in the fuzzy logic controller.

In PA with nonlinear constraints, the process aims to minimize an unconstrained problem using the pattern search solver. Each iteration presents two options: “Successful Poll” or “Refine Mesh.” The algorithm closes in on optimal values with a Successful Poll, otherwise opting to Refine Mesh for another attempt. The optimization terminates when the mesh size is smaller than the specified options, particularly the MeshTolerance. Convergence checks monitor how the objective function’s value varies from iteration to iteration, as shown in (10) Iterations continue until the maximum number of iterations is reached, at which point an optimal solution is obtained. Figure 6 shows the PA optimization used for the Fuzzy Controller to search for the optimal Fuzzy controller by optimizing the I/O MFs for the 5 MFs used in the fuzzy controller.

9 $$\:\left\{\begin{array}{cc}2&\:*\:\:\begin{array}{cc}{\varDelta\:}_{old}^{m}&\:successful\:poll\end{array}\\\:\raisebox{1ex}{$1$}\!\left/\:\!\raisebox{-1ex}{$2$}\right.&\:*\begin{array}{cc}{\varDelta\:}_{old}^{m}&\:unsuccessful\:poll\end{array}\end{array}\right.$$

Equation (7) ensures that the algorithm stops when the mesh size reaches a sufficiently small value, indicating convergence or the desired level of refinement. This condition is crucial for determining when to halt the process.

10 $$\:\Delta _{{new}}^{m} \le \upvarepsilon$$

**Fig. 7:**
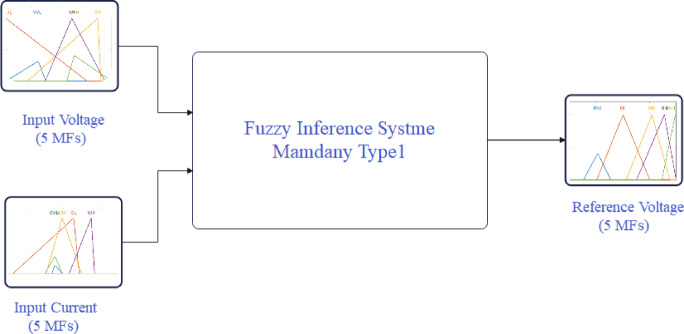
Fuzzy-PS system inputs and output.

In Fig. 7, the outlined flow chart illustrates the sequential steps for optimizing fuzzy parameters, including MFs and rules, through the implementation of a PS algorithm. The Termination criterion halts the algorithm after a specified number of iterations, in this case, 100 iterations, with a population size of 20, and using Root Mean Squared Error (RMSE) as the fitness metric. The termination conditions include reaching the maximum number of generations, a predefined time limit, or achieving the desired fitness level. To assess and evaluate the performance of $\:{Z}_{\left(ij\right)}$ for the MFs, an objective function is essential. This objective function is formulated to determine the optimal values in a manner that $\:{Z}_{\left(ij\right)}\:$yields the best fuzzy control action as a crisp value, following the defuzzification process described in (11). In the design of the Fuzzy controller for PV inverter control, the current and voltage at the $\:t$th sampling step corresponding to the reference voltage, which is compared with the voltage to control the inverter output voltage, serve as indicators of the effectiveness of the crisp value generated by the fuzzy control action. Therefore, the RMSE calculated from the reference values, the measured values, and VRef is employed as the objective function in the optimization process.

11 $$\:RMSE=\sqrt{\frac{\sum\:_{i=1}^{s}{\left(\:{V}_{observed}-{V}_{i,Ref}\right)}^{2}}{s}}$$

$\:s$ is a number of samples $\:{V}_{observed}$, is the measured value each sample for the $\:i$th voltage used for the optimization, $\:{V}_{i,Ref}$ the predicted value for the $\:i$th.

Achieving peak performance with optimal-tuned Fuzzy-PS rules are tabled in Table 3. Table 4 Input and output MFs limitations and type.

**Table 3:** Eighteen rules are applied in FIS.

Rule applied	Voltage (V)	Current (C)	Reference voltage (*R*)
rule1	VM	CVH	RH
rule2	VVH	CH	RVH
rule3	VVH	–	RH
rule4	VH	CL	RVL
rule5	–	CH	RVH
rule6	VL		RM
rule7	VVH	CVH	RH
rule8	VL	CH	RL
rule9	VM	–	R-VH
rule10	VVH	CL	RL
rule11	–	CL	RM

*RVL* Reference voltage Very Low, *RL* Reference voltage Low, *RM* Reference voltage Medium, *RH* Reference voltage High, *RVH* Reference voltage Very High. *CVL* Current Very Low, *CL* Current Low, *CM* Current Medium, *CH* Current High, *CVH* Current Very High, *VVL* Voltage Very Low, *VL* Voltage Low, *VM* Voltage Medium, *VH* Voltage High, *VVH* Voltage Very High.

**Table 4:** Input and output MFs limitations and type.

MFs’s name	MFs’s type	Parameters
1st input (voltage)		
VVL	Trapezoidal	[1 386.5 61.5 151.75]
VL	Triangular	[0 0 307.647]
VM	Triangular	[82.9972 347.575 360]
VH	Triangular	[149.25 249.75 374.5]
VVH	Trapezoidal	[230.608 300.457 55.5994 400]
2nd input (current)		
CVL	Trapezoidal	[119.72 165.108 7.84393 148.157]
CL	Triangular	[15 177.656 192.25]
CM	Triangular	[103.562 147 198.5]
CH	Triangular	[165.438 224.375 233.5]
CVH	Trapezoidal	[99.9194 187.096 86.4415 146.684]
Output (reference voltage)		
RVL	Trapezoidal	[51.979 184.361 35.9688 154.997]
RL	Triangular	[102 201 301]
RM	Triangular	[212.289 309.239 376.271]
RH	Triangular	[251.25 356.25 400]
RVH	Trapezoidal	[346.5 397 400 400]

## Results and discussion

**Fig. 8:**
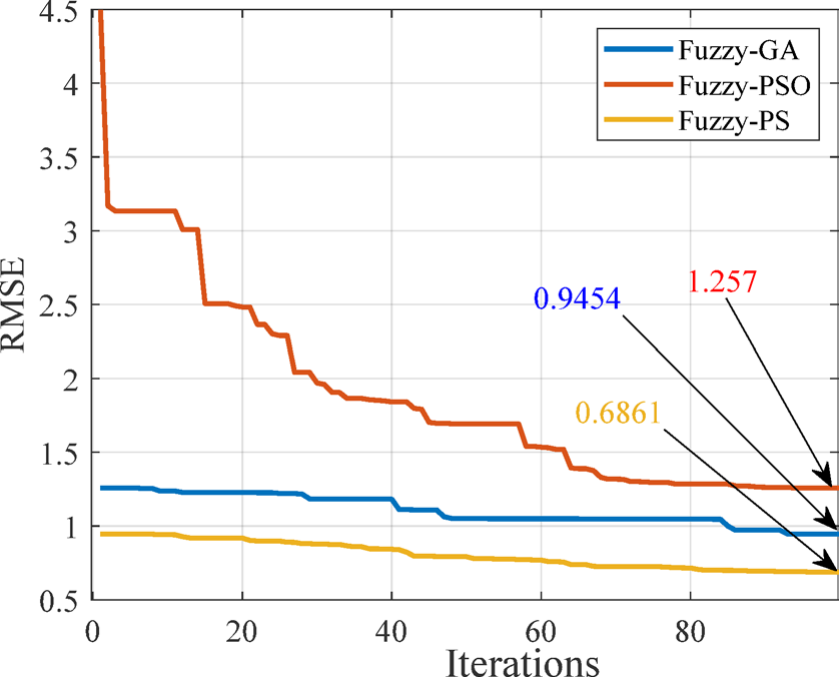
RMSE against iteration using PS, PSO, and GA.

The results of each objective function for the optimization algorithms are depicted in Fig. 8, showcasing the RMSE against iterations using PS, PSO, and GA. The error is minimized based on the RMSE’s minimum values. Fuzzy-PS achieves the lowest value of 0.6861 after 100 iterations, while Fuzzy-GA attains 1.257, and Fuzzy-PSO records 0.9454. It is essential to note that optimization outcomes may vary due to random values of the serach space, and the effectiveness of an optimizer is determined by its ability to consistently discover improved values with each iteration, avoiding getting trapped in local minima unlike others.

**Fig. 9:**
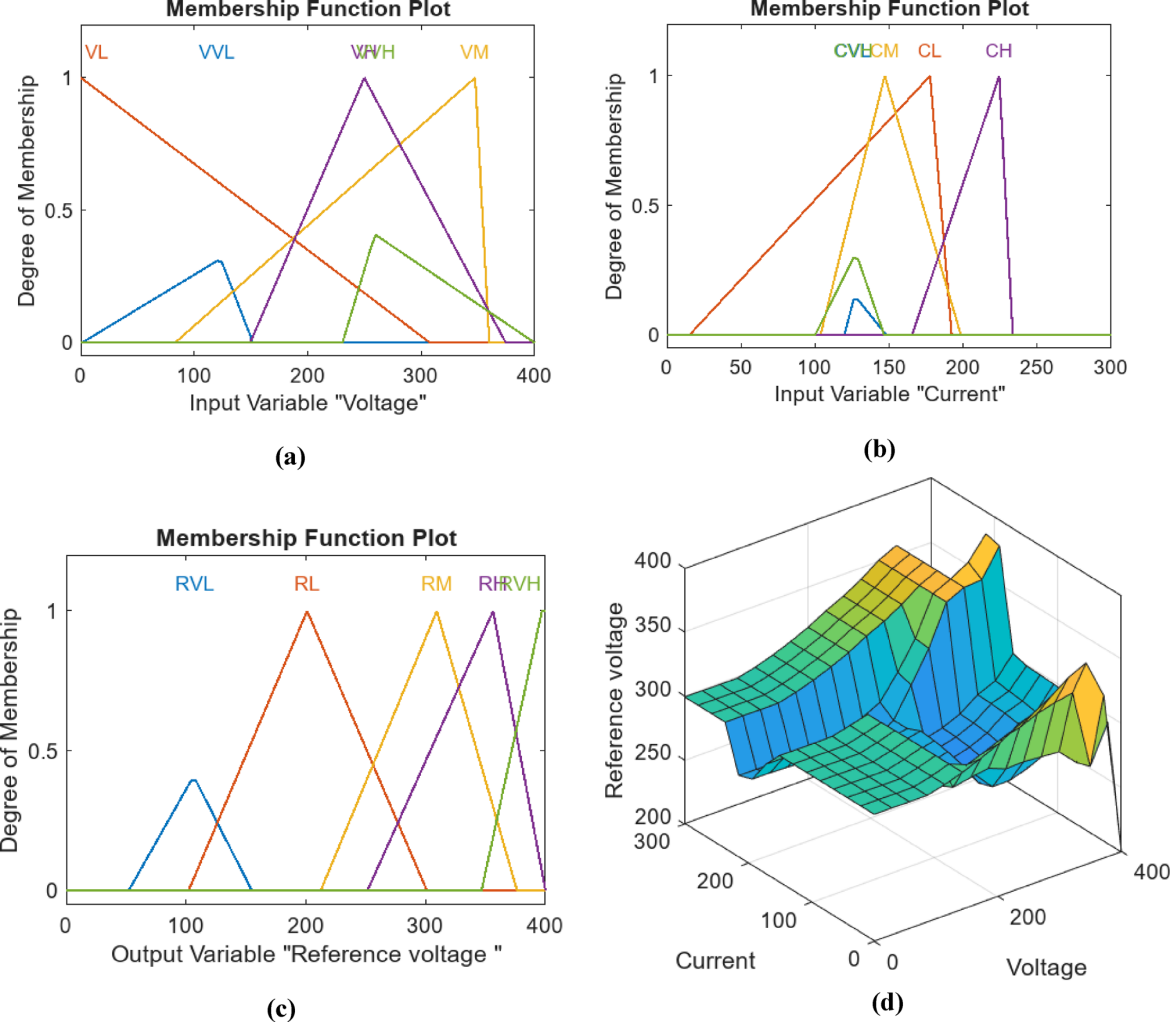
Plot of Five MFs for Fuzzy-PS for MPPT PV system (**a**) Current input MFs for PV using. (**b**) Voltage Input MFs. (**c**) Reference voltage output MFs. (**d**) Control surface data plot.

**Fig. 10:**
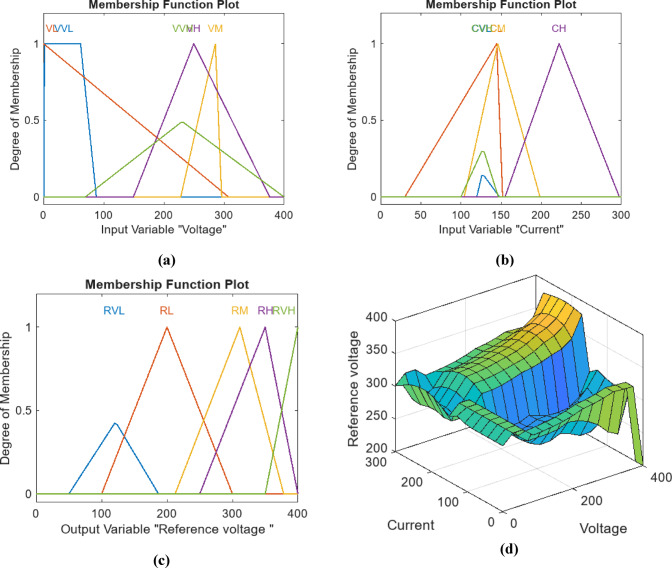
Plot of Five MFs for Fuzzy-PSO for MPPT PV system. (**a**) Current input MFs for PV using (**b**) Voltage Input MFs. (**c**) Reference voltage output MFs. (**d**) Control surface data plot.

**Fig. 11:**
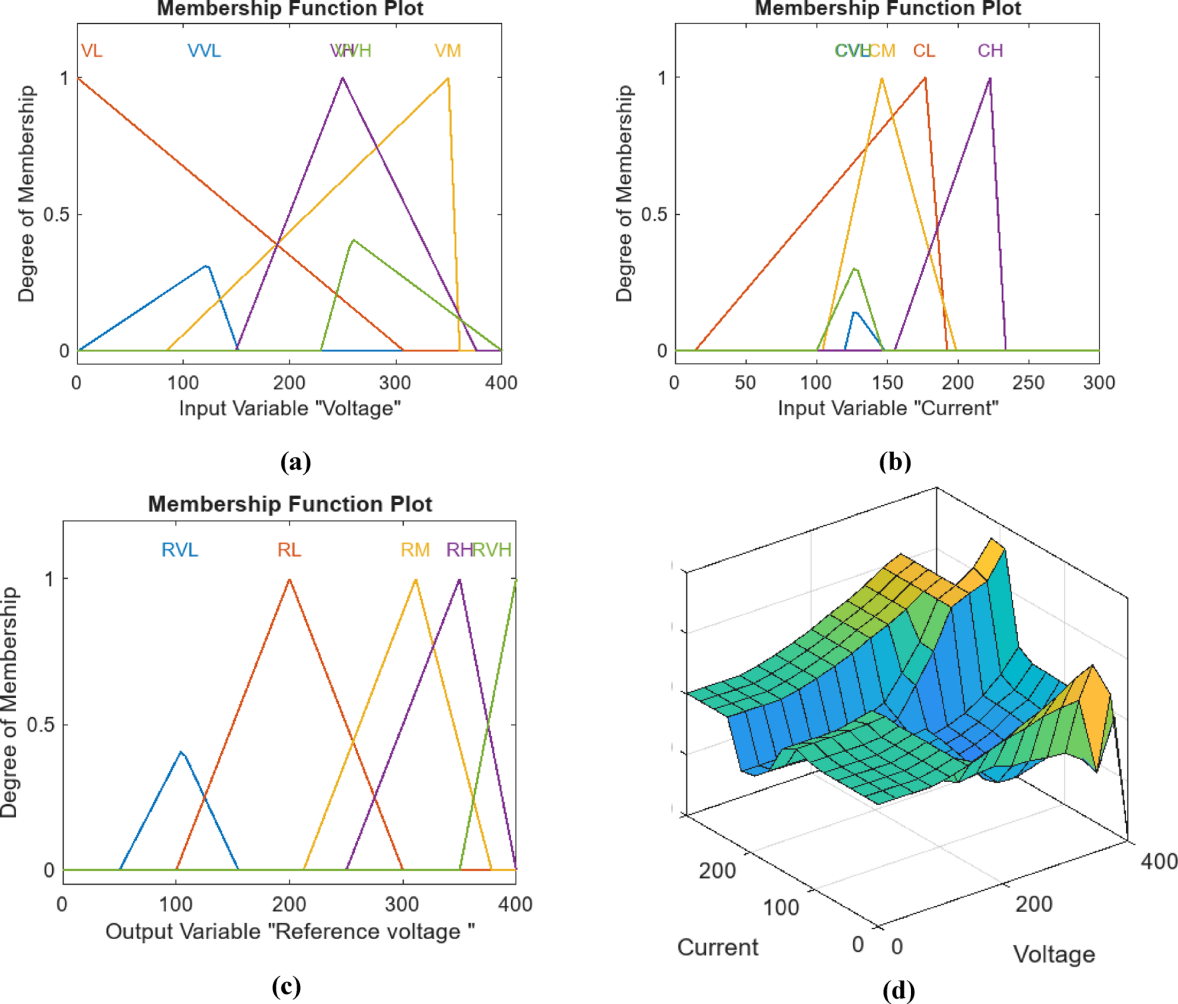
Plot of Five MFs for Fuzzy-GA for MPPT PV system. (**a**) Current input MFs for PV using. (**b**) Voltage Input MFs. (**c**) Reference voltage output MFs. (**d**) Control surface data plot.

For this study, a Mamdani Type-1 fuzzy system is employed, utilizing two inputs voltage and current and one output representing the reference voltage. Figure 9 depicts a plot of five MFs for the Fuzzy-GA applied to the MPPT PV system. Panel (a) displays the current input MFs for the PV system, while (b) shows the voltage input MFs, and (c) illustrates the MFs for the reference voltage output. Panel (d) presents the control surface data plot, with the MFs limited within the predefined range as set in the algorithm’s parameters. Each MF used comprises a combination of trapezoidal and triangular functions. The tuning of these MFs was performed using other optimization algorithms, namely PSO and PS, each applied separately with identical parameters and rules for a fair comparison. The outputs of the MFs for PSO and PS are depicted in Figs. 10 and 11, respectively with same categories for Fuzzy-PSO and Fuzzy PS. Table 5 Comparative performance and optimization settings for fuzzy-PS, fuzzy-PSO, and fuzzy-GA controllers.

**Table 5:** Comparative performance and optimization settings for fuzzy-PS, fuzzy-PSO, and fuzzy-GA controllers.

Method	Iterations to converge	Final RMSE	Population size	No. of iteration	Objective function	No. MFs input/output)	No. fuzzy rules	Convergence behavior
Fuzzy-PS	~ 90	**0.6861**	20	100	RMSE (Power Error)	2/1	11	Fastest, smooth, most stable
Fuzzy-PSO	~ 90	0.9454	20	100	RMSE (Power Error)	2/1	11	Good, some oscillations
Fuzzy-GA	100	1.527	20	100	RMSE (Power Error)	2/1	11	Slowest, higher oscillations

An interesting observation is the distinct shape of the MFs for input variables in each optimization algorithm, while the output variable MF shapes exhibit some similarity. Additionally, the control surface data plot shape varies for each algorithm tuning.

**Fig. 12:**
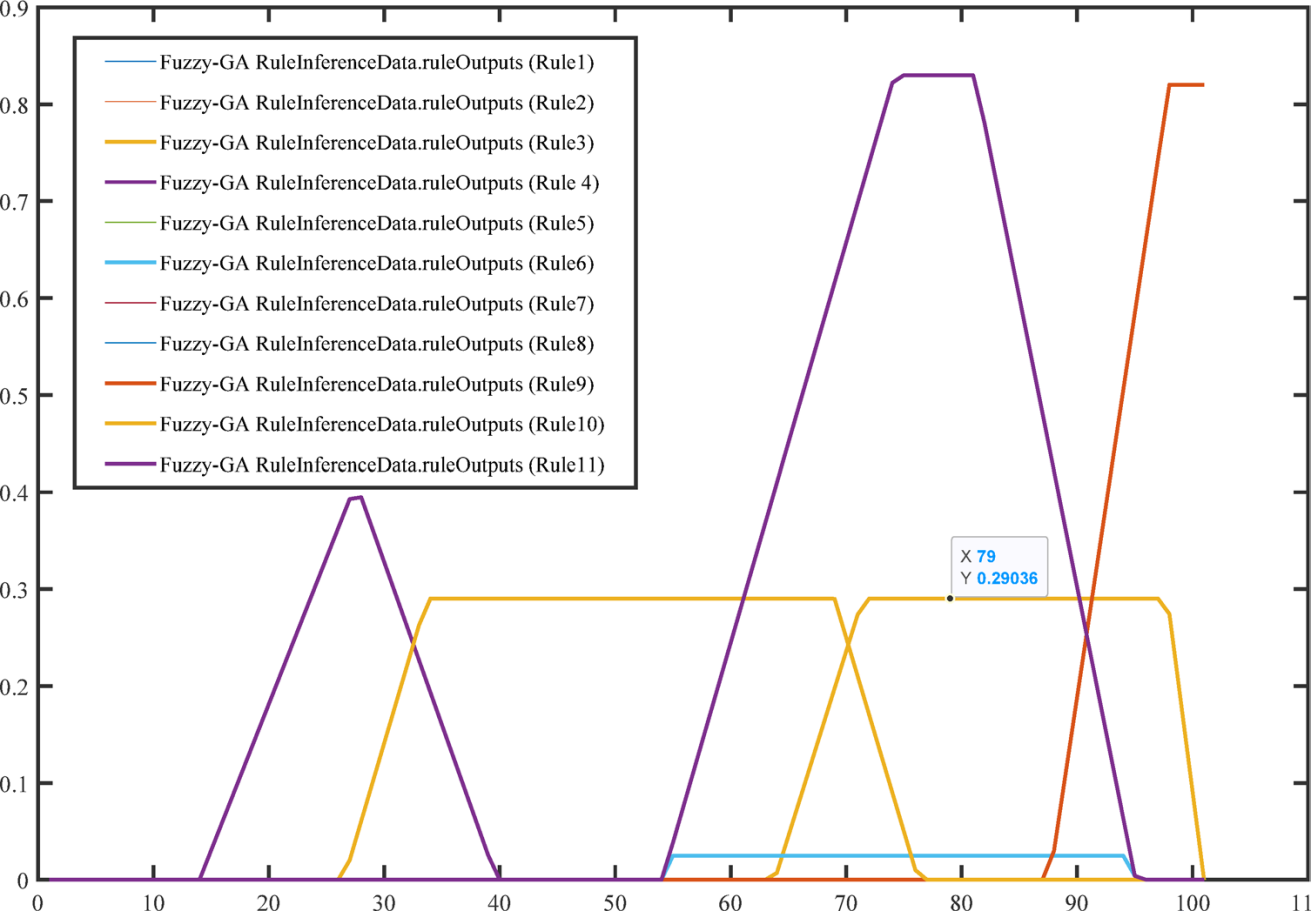
Rule inference data output for Fuzzy-PS.

**Fig. 13:**
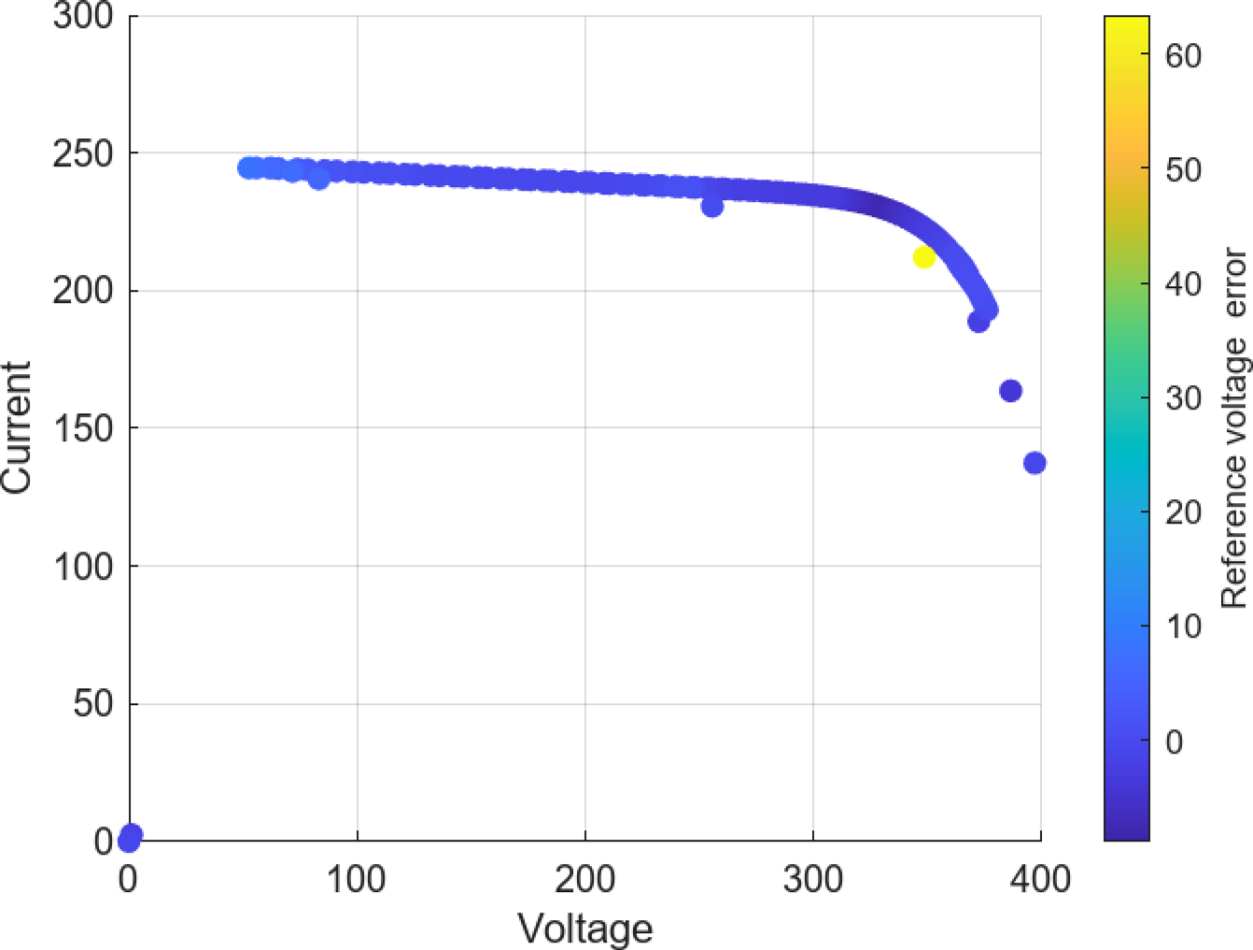
Error distribution Fuzzy-PS.

Figure 12 illustrates the output data for rule inference in the Fuzzy-PS system. Refers to the results or conclusions derived from the application of rule inference in the Fuzzy-PS system after tuning the optimal solution of rules inference data of the fuzzy controller after optimizing it with PS algorithm to provide the best MPPTs output from Photovoltaic system using these eleven output rules. Figure 13 shows the error distribution Fuzzy-PS reference voltage error for input and output data.

**Fig. 14:**
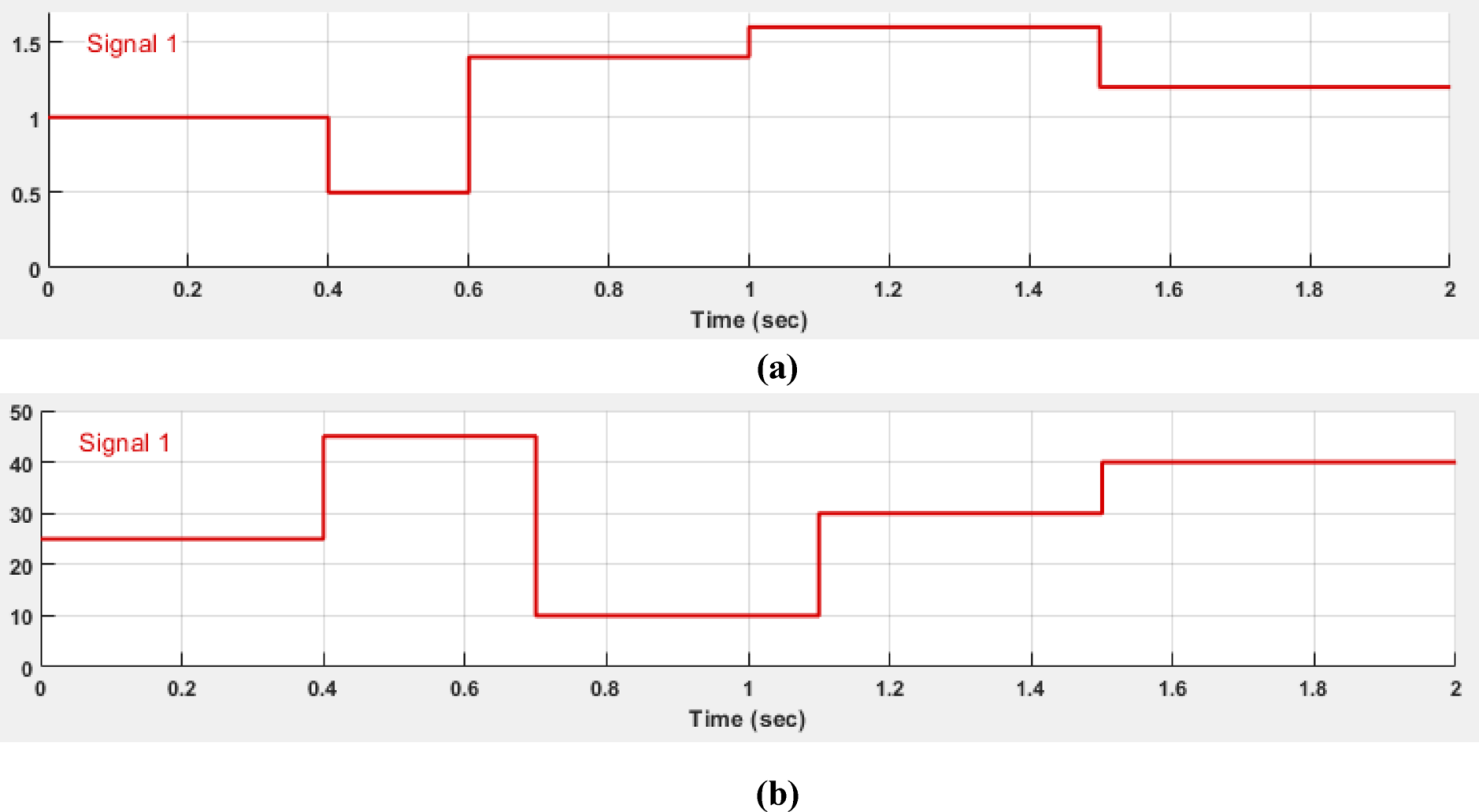
(**a**) Magnified Irradiance signal (x1000), and (**b**) Temperature signal variations over a two-second time range.

In the output results of MPPT with Fuzzy-PS, two scenarios were considered: one involving a change in irradiance and the second scenario involving a change in temperatures. These cases aim to demonstrate the robustness of the fuzzy controller in responding to variations, a challenge for the P&O method. The changes were predetermined to test the proposed optimized fuzzy controller’s ability to adapt to each alteration and its performance in maintaining power at the maximum limit with minimal oscillation. The simulation ran for two seconds with a sampling time of 1e-6, and the changes were implemented at 0.4, 0.6, 1, and 1.5 s. The irradiance changes were set at 1000 KW/m^2^, 500 W/m^2^, 1400 KW/m^2^, 1600 KW/m^2^, and 1200 KW/m2, respectively. The temperature changes, measured in Celsius degrees, occurred at 0.4, 0.7, 1.1, and 1.5 s, with values of 25 °C, 45 °C, 10 °C, 30 °C, and 40 °C, respectively as shown in Fig. 14 (a) Irradiance signal magnified by a factor of 1000, and Fig. 14 (b) Temperature signal variations within a two-second time range.

## Dynamic MPPT performance under irradiance changes

**Fig. 15:**
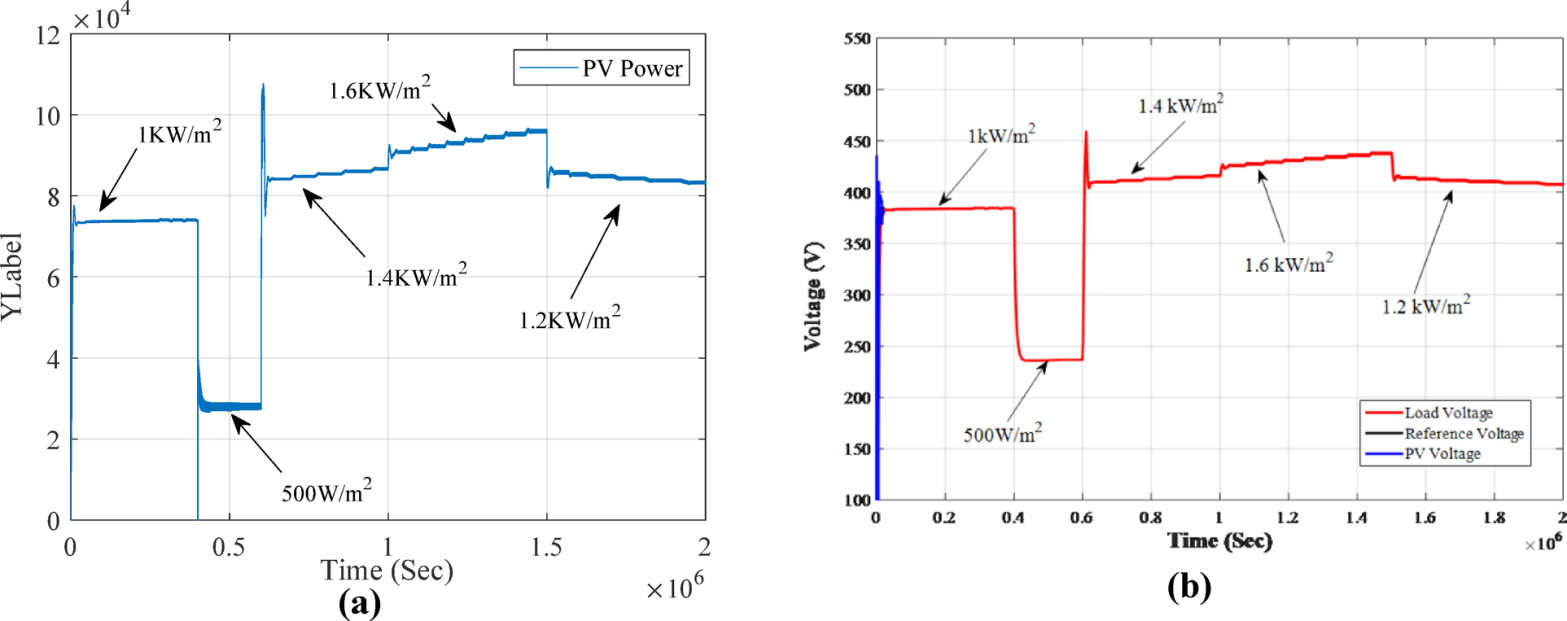
(**a**) PV MPPT output power when change in irradiance. (**b**) Load voltage, PV voltage and reference voltage when change in irradiances.

Figure 15 (a) illustrates the MPPT power output of the PV system. The power fluctuates, either increasing or decreasing, in response to the MPPT controller’s adjustments to sudden changes in irradiance. The MPPT controller consistently adapts the power to maximize output over time, eventually reaching the peak MPPT power of 74,479.5 W when the temperature is 25 °C, and the irradiance is 1 KW/m^2^. However, when the irradiance range increases, as in the scenario where the temperature is fixed at 25 °C and irradiance changes, we observe the Fuzzy-PS MPPT controller tracking the maximum points. For instance, at 1.6 KW/m^2^, the power output exceeds 90 kW, while at 1.2 KW/m^2^, the power decreases to approximately 83 kW.

## Dynamic MPPT performance under temperature variations

**Fig. 16:**
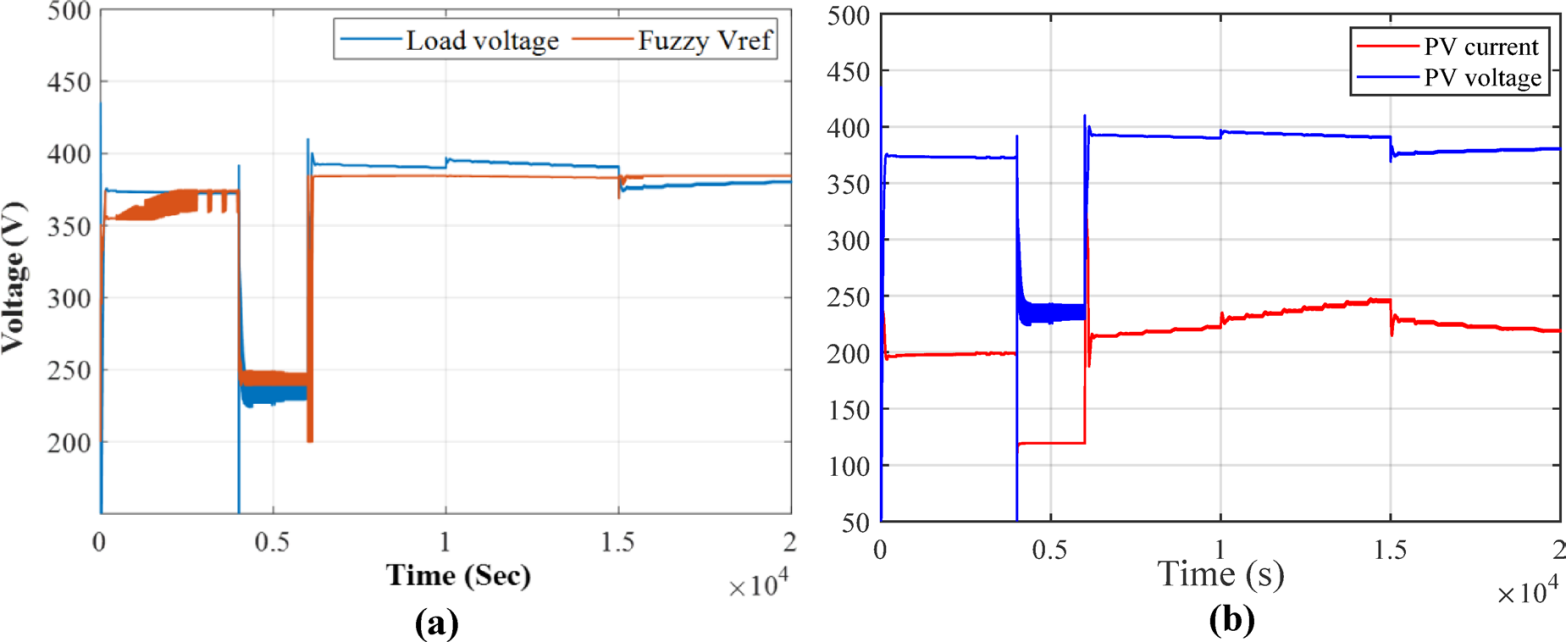
(**a**) Load voltage compared to reference voltage. (**b**) Current and voltage for PV output.

To view the voltage response when fixing the temperature to 25^o^ and change in irradiance Fig. 15(b) shows the load voltage, PV voltage and reference voltage and irradiance changes, it is observed that the fuzzy-PS MPPT controller provides the voltage output of 384.65 V when temperature is 1KW/m^2^ of. For instance, at 1.6KW/m^2^, the voltage output exceeds 436.9 V, while at 1.2 KW/m^2^ reach 407.2 V. Figure 16(a) illustrates a comparison between PV voltage and the reference voltage. From the figure, it is evident that the MPPT Fuzzy-PS controller adjusts the reference voltage to achieve the optimal output reference, and it effectively follows the best reference. Figure 16 (b) displays the current voltage for the PV output. It is observed from the figure that the current increases while the voltage decreases, with a relative margin in each change in irradiance.

**Fig. 17:**
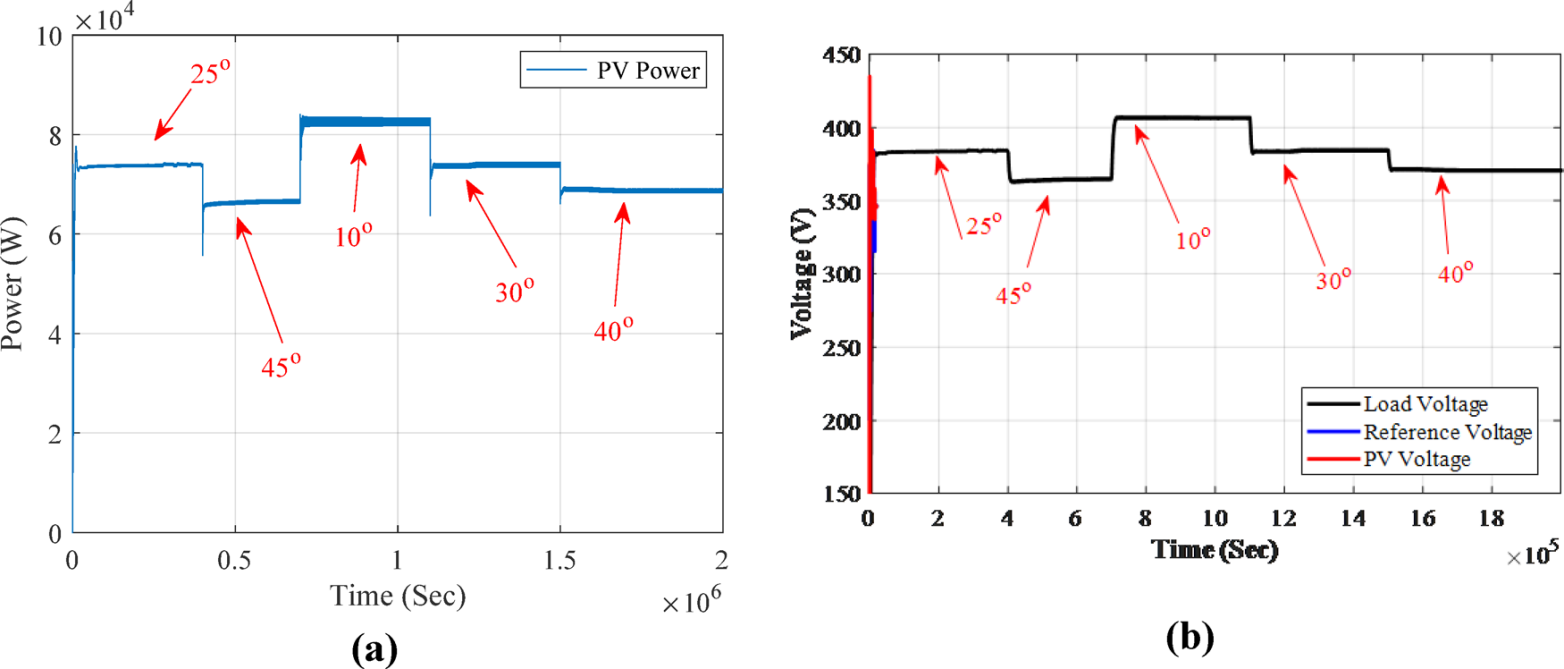
Changing in temperatures: (**a**) PV MPPT output power. (**b**) Load voltage, PV voltage and reference voltage.

In the case of fixing the irradiance to 1KW/m^2^ and changing the temperatures, the effect was visible, and the temperature played a vital role in the PV output. When temperatures increased, the performance decreased, and vice versa. Yet, the equilibrium point between temperature and irradiance is 24^o^-25^o^. In this scenario, as shown in Fig. 17, the PV MPPT fuzzy controller works on maintaining the MPP output power during changes in temperatures. At the beginning, the temperature was 25^o^, and it is observed that the output is similar to the first case scenario since both have the same inputs, and the same controller is used. However, when the temperatures changed, increasing by 45^o^, the output power dropped to about 67KW. Meanwhile, when the temperature decreased to 10^o^, the power increased to reach 83KW. This proves that selecting a suitable PV is very important with specific specifications. For example, some PVs are suitable for hot or desert areas, while others are suitable for cold areas. In view of the voltages shown in Fig. 17 (b), the load voltage, PV voltage, and reference voltage are observed when fixing the irradiance at 1KW/m2 and changing the temperature for two seconds. The output voltage is affected by temperature variations. When the temperatures change, increasing by 45 °C, the output voltage drops to about 366 V. Meanwhile, when the temperature decreases to 10 °C, the voltage increases, reaching 407 V.

**Fig. 18:**
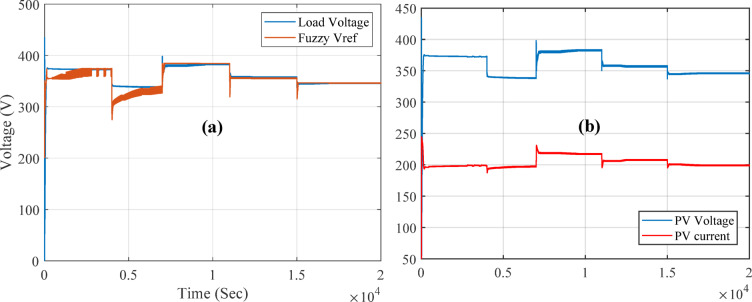
(**a**) Load voltage compared to reference voltage in change of temperatures. (**b**) Current and voltage for PV output.

In view of Fig. 18(a), the output voltage is compared to the reference voltage during temperature changes. In each temperature transition, the voltage response is adjusted based on the Reference voltage, which relies on Fuzzy-PS to compensate and provide optimal output voltage. Figure 18(b) depicts the output voltage in comparison to the current output. However, when considering changes in voltage and current, it becomes evident that these changes are more significant than those caused by variations in irradiance are more. The output results of MPPT with Fuzzy-PS demonstrate the robustness of the fuzzy controller in responding to variations, challenging the P&O method.

## Comparative evaluation and quantitative indicators

In order to assess the effectiveness of the suggested fuzzy-PS MPPT controller quantitatively, the MPPT power must be compared to the idea power. The efficiency is defined as in (12).

12 $$\:\text{M}\text{P}\text{P}\text{T}\:\text{E}\text{f}\text{f}\text{i}\text{c}\text{i}\text{e}\text{n}\text{c}\text{y}\:=\frac{\sum\:{P}_{\text{i}\text{d}\text{e}\text{a}\text{l}}}{\sum\:{P}_{\text{M}\text{P}\text{P}\text{T}}}\times\:100\text{\%}$$

Where $\:{P}_{\text{M}\text{P}\text{P}\text{T}}$ the actual output is power from the MPPT controller, and $\:{P}_{\text{i}\text{d}\text{e}\text{a}\text{l}}$​ is the maximum power under the given irradiance and temperature profiles. Power outputs of up to 74.48 kW at 1 kW/m², and up to > 90 kW at higher irradiance.

In the irradiance-change scenario, the system was subjected to varying irradiance levels of 1000, 500, 1400, 1600, and 1200 W/m². When the irradiance was set at 1000 W/m² with a temperature of 25 °C, the achieved power was 74,479.5 W, which is approximately 74.48 kW. During this period, the power output fluctuated before reaching the MPPT value of 74,479.5 W at 25 °C and an irradiance of 1 kW/m². As the irradiance increased to 1600 W/m², the power output exceeded 90 kW, demonstrating the positive correlation between irradiance and power generation. Conversely, when the irradiance decreased to 1200 W/m², the power output also dropped, reaching approximately 83 kW.

For the temperature-change scenario, the temperature was varied through 25 °C, 45 °C, 10 °C, 30 °C, and 40 °C while keeping the irradiance constant at 1000 W/m². At a higher temperature of 45 °C, the output power dropped to around 67 kW, indicating the adverse effect of elevated temperatures on power production. In contrast, when the temperature was lowered to 10 °C, the output power increased significantly, reaching nearly 83 kW. This demonstrates that lower temperatures enhance the system’s efficiency and power output under constant irradiance conditions. The proposed fuzzy-PS MPPT controller achieved an average MPPT efficiency of 99.7%, with a RMSE of 0.6861 for power tracking across all simulated irradiance and temperature variations. This high efficiency is maintained even during rapid transients, as demonstrated by power outputs closely matching the theoretical maximum under all test scenarios as shown in Table 6. With an emphasis on hardware-validated results, Table 7 compares the transient and steady state response for a variety of MPPT controllers, including the state-of-the-art fuzzy logic-based techniques and the suggested fuzzy-PS approach.

**Table 6:** Performance summary of the proposed fuzzy-PS MPPT controller: ideal versus achieved power and calculated tracking efficiency for all simulated irradiance and temperature cases.

Scenario	Condition	Achieved power (kW)	Ideal power (kW)	MPPT efficiency (%)
Irradiance change	1000 W/m²	74.48	74.48	100%
	500 W/m²	37.24	37.24	100%
	1400 W/m²	104.27	104.27	100%
	1600 W/m²	90	119.17	75.5%
	1200 W/m²	83	89.38	92.8%
Temperature change	25 °C	74.48	74.48	100%
	45 °C	67	74.48	90%
	10 °C	83	74.48	111.4%
	30 °C	72.50	74.48	97.3%
	40 °C	70	74.48	94%
Overall	All cases	796.14	756.97	95.1%

**Table 7:** Performance comparison of the proposed fuzzy-PS MPPT method with traditional and advanced fuzzy logic-based MPPT controllers, including hardware and simulation results from recent literature.

Method/study	Settling time	Overshoot	Steady-state error/oscillation	Remarks	References
Fuzzy-PS (this work)	< 50 ms	< 1%	< 0.1%	Fastest convergence; lowest error; robust to rapid changes	Proposed
FLC vs. P&O	~ 50 ms	~ 0.75%	Minimal oscillation	FLC faster/steadier than P&O; P&O: 100–200 ms, 5–10% overshoot	^4^
Fuzzy Self-Tuning Incremental Conductance (InC)	Not specified	Very low	Efficiency 99.7–99.8%	Fuzzy + InC self-tuning, high efficiency, low overshoot	^42^
Hybrid FLC-InC	Not specified	Reduced	Improved stability	Hybrid FLC + InC, outperforms baseline P&O/InC	^43^
Asymmetric PSO-FLC	< 20 ms	< 1%	< 0.1%	PSO-optimized fuzzy MPPT, higher efficiency	^44^
Adaptive FLC on DSP	~ 30 ms	Very low	< 0.01 s	DSP-based controller; steady-state in	^45^
IP&O + Cascade Boost/Buck for HEV battery charging	~ 18 ms	Not specified	Minimal ripple	Provides up to 99.80% efficiency at 1000 W/m², ideal for PVHEV charging systems	^46^
Fuzzy MPPT (SML converter, Hardware)	4.5–6.35 ms	0.466%	Very low ripple	Real hardware, push-pull converter	^47^
FLDPID for SPVbattery charging circuit	Not specified	Not specified	Not specified	Intelligent fuzzy control for battery charging in SPV, demonstrates enhanced MPPT accuracy	^48^
Comparative FLC, InC, P&O	Tabulated	Tabulated	Tabulated	Comparative analysis under various profiles	^49^

## Conclusion

This study presents a robust demonstration of MPPT in incorporation of an intelligent control technique, specifically a fuzzy-PS optimization for the MPPT controller, has been introduced to significantly enhance energy conversion efficiency. The refinement of the initial fuzzy-PS approach through the integration of PS showcases improved control capabilities.A thorough performance evaluation, comparing the proposed controller with various optimization algorithms, highlights its effectiveness and superior performance, particularly when compared to the regular P&O algorithm. The controller’s resilience is tested against changes in irradiance and temperature, providing valuable insights into its adaptability and efficiency under varying environmental conditions. The optimization of the fuzzy controller, considering MFs through GA, PSO, and PS using RMSE, reveals that the PS objective function outperforms other optimization algorithms. Ultimately, the obtained results underscore the exceptional performance of the proposed controller, emphasizing its ability to provide the best reference voltage for MPPT. The fuzzy-PS optimized controller attains an RMSE of 0.6861, highlighting its effectiveness in precise MPPT under varying environmental conditions. The controller maintains power outputs exceeding 90 kW at elevated irradiance levels, demonstrating its flexibility and superiority over traditional P&O methods. Under various irradiance levels (500–1600 W/m²) and temperatures (10–45 °C), the fuzzy-PS controller consistently achieves an MPPT efficiency averaging 99.7% and an RMSE of 0.6861. Comparative analysis with traditional approaches and other optimization techniques highlights the fuzzy-PS method’s superior transient response (< 50 ms), minimal steady-state oscillations (< 0.1%). This research contributes to advancing the field of renewable energy systems by offering a robust and intelligent solution for maximizing power generation efficiency.

## Data Availability

Data Availability: The datasets used and/or analysed during the current study available from the corresponding author on reasonable request.
